# 2-[(2-Hydr­oxy-1-naphth­yl)methyl­ene­amino]-1,3-benzothia­zole

**DOI:** 10.1107/S1600536808029644

**Published:** 2008-09-20

**Authors:** Liansheng Cui, Handong Yin, Minglei Yang, Li Quan, Daqi Wang

**Affiliations:** a College of Chemistry and Chemical Engineering, Liaocheng University, Shandong 252059, People’s Republic of China

## Abstract

In the title compound, C_18_H_12_N_2_OS, the dihedral angle between the mean planes of the aromatic ring systems is 7.26 (8)°. There is an intra­molecular O—H⋯N hydrogen bond, forming a six-membered ring. In the crystal structure, inter­molecular C—H⋯N hydrogen bonds link the mol­ecules into a one-dimensional chain.

## Related literature

For general background, see: Lindoy *et al.* (1976 ▶).
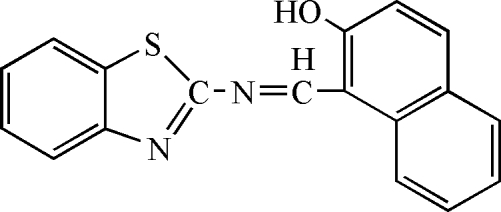


## Experimental

### 

#### Crystal data

C_18_H_12_N_2_OS
*M* *_r_* = 304.36
Monoclinic, 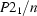

*a* = 9.7439 (13) Å
*b* = 15.1506 (16) Å
*c* = 9.8082 (14) Å
β = 101.788 (2)°
*V* = 1417.4 (3) Å^3^
*Z* = 4
Mo *K*α radiation
μ = 0.23 mm^−1^
*T* = 293 (2) K
0.29 × 0.24 × 0.16 mm

#### Data collection

Siemens SMART CCD area-detector diffractometer
Absorption correction: multi-scan (*SADABS*; Sheldrick, 1996 ▶) *T*_min_ = 0.936, *T*_max_ = 0.964
6948 measured reflections
2493 independent reflections
1492 reflections with *I* > 2σ(*I*)
*R*_int_ = 0.048

#### Refinement

*R*[*F* ^2^ > 2σ(*F* ^2^)] = 0.046
*wR*(*F* ^2^) = 0.122
*S* = 1.06
2493 reflections
199 parameters
H-atom parameters constrained
Δρ_max_ = 0.22 e Å^−3^
Δρ_min_ = −0.27 e Å^−3^

### 

Data collection: *SMART* (Siemens, 1996 ▶); cell refinement: *SAINT* (Siemens, 1996 ▶); data reduction: *SAINT*; program(s) used to solve structure: *SHELXS97* (Sheldrick, 2008 ▶); program(s) used to refine structure: *SHELXL97* (Sheldrick, 2008 ▶); molecular graphics: *SHELXTL* (Sheldrick, 2008 ▶); software used to prepare material for publication: *SHELXTL*.

## Supplementary Material

Crystal structure: contains datablocks I, global. DOI: 10.1107/S1600536808029644/sg2264sup1.cif

Structure factors: contains datablocks I. DOI: 10.1107/S1600536808029644/sg2264Isup2.hkl

Additional supplementary materials:  crystallographic information; 3D view; checkCIF report

## Figures and Tables

**Table 1:** Hydrogen-bond geometry (Å, °)

*D*—H⋯*A*	*D*—H	H⋯*A*	*D*⋯*A*	*D*—H⋯*A*
C16—H16⋯N1^i^	0.93	2.63	3.389 (4)	140
O1—H1⋯N2	0.82	1.84	2.576 (2)	148

Symmetry code: (i) 
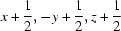
.

**Fig. 1.**
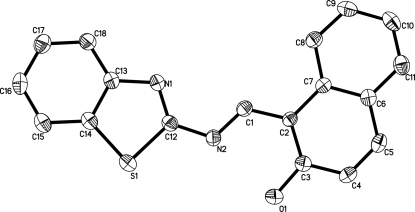
The structure of the title compound, showing 30% probability displacement ellipsoids and the atom-numbering scheme.

**Fig. 2.**
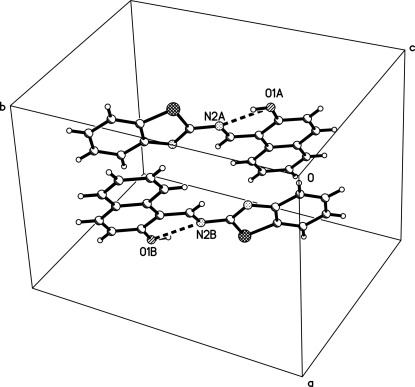
The crystal packing of the title compound, viewed approximately along the c axis.

## supplementary crystallographic information

### Comment

Schiff bases are known to be important due to their applications in the preparation of dyes, liquid crystals and powerful corrosion inhibitors. Further more, they are used in the mechanism of many biochemical processes (Lindoy *et al.*, 1976). We report here the synthesis and crystal structure of the title compound, a new Schiff base compound.

The molecular structure of the title compound is shown in Fig. 1. This compound is nonplanar, with a dihedral angle of 7.39 (5)° between the two benzene rings. There exists an intramolecular O—H···N hydrogen bond, forming a six-membered ring. An E configuration with respect to the C?N bond is shown by the molecule, with a C—N?C—C torsoin angle of 178.8 (2)°.

As seen in Fig.2, the molecules are linked into a one-dimensional chain by intermolecular C—H···N hydrogen bonds (Table 1).

### Experimental

A mixture of 2-aminobenzothiazole (1 mmol) and 2-hydroxy-1-naphthaldehyde (1 mmol) in absolute ethanol (15 ml) was heated under reflux with stirring for 5 h and then filtered. The resulting clear orange solution was diffused diethyl ether vapor at room temperature for 16 days, after which large orange block-shaped crystals of the title complex suitable for X-ray diffraction analysis were obtained.

### Refinement

All H-atoms were positioned geometrically and refined using a riding model, with C—H = 0.96 Å (methylene) or 0.93 Å (aromatic), 0.82 Å (hydroxyl) and *U*_iso_(H) =1.2*U*_eq_(C).

### Crystal data

**Table:**

C_18_H_12_N_2_OS	*F*(000) = 632
*M**_r_* = 304.36	*D*_x_ = 1.426 Mg m^−^^3^
Monoclinic, *P*2_1_/*n*	Mo *K*α radiation, λ = 0.71073 Å
Hall symbol: -P 2yn	Cell parameters from 1399 reflections
*a* = 9.7439 (13) Å	θ = 2.5–24.1°
*b* = 15.1506 (16) Å	µ = 0.23 mm^−^^1^
*c* = 9.8082 (14) Å	*T* = 293 K
β = 101.788 (2)°	Block, orange
*V* = 1417.4 (3) Å^3^	0.29 × 0.24 × 0.16 mm
*Z* = 4	

### Data collection

**Table:**

Siemens SMART CCD area-detector diffractometer	2493 independent reflections
Radiation source: fine-focus sealed tube	1492 reflections with *I* > 2σ(*I*)
graphite	*R*_int_ = 0.048
φ and ω scans	θ_max_ = 25.0°, θ_min_ = 2.5°
Absorption correction: multi-scan (SADABS; Sheldrick, 1996)	*h* = −6→11
*T*_min_ = 0.936, *T*_max_ = 0.964	*k* = −18→16
6948 measured reflections	*l* = −10→11

### Refinement

**Table:**

Refinement on *F*^2^	Primary atom site location: structure-invariant direct methods
Least-squares matrix: full	Secondary atom site location: difference Fourier map
*R*[*F*^2^ > 2σ(*F*^2^)] = 0.046	Hydrogen site location: inferred from neighbouring sites
*wR*(*F*^2^) = 0.122	H-atom parameters constrained
*S* = 1.06	*w* = 1/[σ^2^(*F*_o_^2^) + (0.0495*P*)^2^ + 0.1122*P*] where *P* = (*F*_o_^2^ + 2*F*_c_^2^)/3
2493 reflections	(Δ/σ)_max_ = 0.001
199 parameters	Δρ_max_ = 0.22 e Å^−^^3^
0 restraints	Δρ_min_ = −0.27 e Å^−^^3^

### Special details

**Table:**

Geometry. All e.s.d.'s (except the e.s.d. in the dihedral angle between two l.s. planes) are estimated using the full covariance matrix. The cell e.s.d.'s are taken into account individually in the estimation of e.s.d.'s in distances, angles and torsion angles; correlations between e.s.d.'s in cell parameters are only used when they are defined by crystal symmetry. An approximate (isotropic) treatment of cell e.s.d.'s is used for estimating e.s.d.'s involving l.s. planes.
Refinement. Refinement of *F*^2^ against ALL reflections. The weighted *R*-factor *wR* and goodness of fit *S* are based on *F*^2^, conventional *R*-factors *R* are based on *F*, with *F* set to zero for negative *F*^2^. The threshold expression of *F*^2^ > σ(*F*^2^) is used only for calculating *R*-factors(gt) *etc*. and is not relevant to the choice of reflections for refinement. *R*-factors based on *F*^2^ are statistically about twice as large as those based on *F*, and *R*- factors based on ALL data will be even larger.

### Fractional atomic coordinates and isotropic or equivalent isotropic displacement parameters (Å^2^)

**Table:**

	*x*	*y*	*z*	*U*_iso_*/*U*_eq_	
N1	0.1366 (2)	0.12262 (16)	1.0288 (2)	0.0448 (6)	
N2	0.2042 (2)	−0.00510 (15)	0.9113 (2)	0.0411 (6)	
O1	0.2722 (2)	−0.14394 (13)	0.7886 (2)	0.0557 (6)	
H1	0.2814	−0.1033	0.8450	0.084*	
S1	0.39272 (8)	0.06918 (6)	1.12063 (8)	0.0516 (3)	
C1	0.0880 (3)	−0.00369 (18)	0.8179 (3)	0.0392 (7)	
H1A	0.0245	0.0416	0.8218	0.047*	
C2	0.0532 (3)	−0.06771 (18)	0.7105 (3)	0.0345 (7)	
C3	0.1482 (3)	−0.13637 (19)	0.6999 (3)	0.0412 (7)	
C4	0.1156 (3)	−0.20068 (19)	0.5945 (3)	0.0474 (8)	
H4	0.1790	−0.2457	0.5887	0.057*	
C5	−0.0075 (3)	−0.19716 (19)	0.5022 (3)	0.0468 (8)	
H5	−0.0275	−0.2408	0.4343	0.056*	
C6	−0.1079 (3)	−0.12922 (18)	0.5045 (3)	0.0386 (7)	
C7	−0.0773 (3)	−0.06359 (18)	0.6093 (3)	0.0340 (7)	
C8	−0.1789 (3)	0.00355 (18)	0.6076 (3)	0.0435 (8)	
H8	−0.1626	0.0479	0.6746	0.052*	
C9	−0.3009 (3)	0.0043 (2)	0.5089 (3)	0.0520 (9)	
H9	−0.3657	0.0493	0.5100	0.062*	
C10	−0.3298 (3)	−0.0610 (2)	0.4072 (3)	0.0536 (9)	
H10	−0.4131	−0.0598	0.3409	0.064*	
C11	−0.2348 (3)	−0.1267 (2)	0.4058 (3)	0.0499 (8)	
H11	−0.2542	−0.1706	0.3384	0.060*	
C12	0.2275 (3)	0.06286 (19)	1.0103 (3)	0.0407 (7)	
C13	0.1972 (3)	0.18005 (19)	1.1355 (3)	0.0395 (7)	
C14	0.3359 (3)	0.16080 (19)	1.1995 (3)	0.0417 (7)	
C15	0.4076 (3)	0.2139 (2)	1.3077 (3)	0.0507 (8)	
H15	0.5000	0.2018	1.3502	0.061*	
C16	0.3372 (4)	0.2847 (2)	1.3493 (3)	0.0535 (9)	
H16	0.3828	0.3205	1.4214	0.064*	
C17	0.1997 (3)	0.3034 (2)	1.2859 (3)	0.0526 (8)	
H17	0.1551	0.3517	1.3162	0.063*	
C18	0.1283 (3)	0.2523 (2)	1.1798 (3)	0.0474 (8)	
H18	0.0360	0.2654	1.1380	0.057*	

### Atomic displacement parameters (Å^2^)

**Table:**

	*U*^11^	*U*^22^	*U*^33^	*U*^12^	*U*^13^	*U*^23^
N1	0.0401 (15)	0.0450 (16)	0.0464 (15)	−0.0018 (12)	0.0022 (12)	−0.0040 (13)
N2	0.0439 (15)	0.0404 (16)	0.0376 (14)	−0.0009 (11)	0.0051 (11)	−0.0026 (12)
O1	0.0471 (14)	0.0532 (14)	0.0624 (14)	0.0129 (10)	0.0010 (11)	−0.0064 (11)
S1	0.0419 (5)	0.0566 (6)	0.0521 (5)	0.0031 (4)	−0.0004 (4)	−0.0015 (4)
C1	0.0415 (18)	0.0363 (18)	0.0411 (17)	0.0000 (13)	0.0114 (14)	0.0002 (14)
C2	0.0372 (17)	0.0296 (16)	0.0380 (16)	−0.0015 (13)	0.0111 (13)	−0.0003 (14)
C3	0.0397 (19)	0.0419 (19)	0.0424 (17)	−0.0002 (14)	0.0094 (14)	0.0024 (15)
C4	0.051 (2)	0.0388 (19)	0.055 (2)	0.0061 (14)	0.0168 (16)	−0.0046 (16)
C5	0.057 (2)	0.040 (2)	0.0458 (18)	−0.0065 (15)	0.0164 (16)	−0.0090 (15)
C6	0.0416 (18)	0.0380 (18)	0.0370 (17)	−0.0061 (14)	0.0097 (14)	0.0037 (14)
C7	0.0389 (17)	0.0304 (16)	0.0355 (15)	−0.0019 (13)	0.0141 (13)	0.0042 (13)
C8	0.0469 (19)	0.0368 (19)	0.0482 (18)	−0.0008 (14)	0.0130 (15)	−0.0016 (14)
C9	0.040 (2)	0.051 (2)	0.063 (2)	0.0078 (15)	0.0045 (16)	0.0094 (18)
C10	0.045 (2)	0.061 (2)	0.0485 (19)	−0.0102 (17)	−0.0051 (15)	0.0084 (18)
C11	0.057 (2)	0.046 (2)	0.0450 (19)	−0.0123 (16)	0.0061 (16)	−0.0008 (16)
C12	0.0388 (18)	0.0401 (18)	0.0422 (17)	−0.0010 (14)	0.0057 (14)	0.0049 (16)
C13	0.0413 (18)	0.0428 (19)	0.0341 (16)	−0.0054 (14)	0.0072 (14)	0.0033 (14)
C14	0.0399 (18)	0.0445 (19)	0.0403 (16)	−0.0066 (14)	0.0069 (14)	0.0080 (15)
C15	0.046 (2)	0.059 (2)	0.0432 (18)	−0.0085 (16)	0.0008 (15)	0.0042 (17)
C16	0.066 (2)	0.049 (2)	0.0438 (19)	−0.0179 (17)	0.0057 (17)	−0.0044 (16)
C17	0.064 (2)	0.042 (2)	0.053 (2)	−0.0047 (16)	0.0144 (17)	0.0001 (16)
C18	0.0431 (19)	0.048 (2)	0.0494 (19)	−0.0001 (15)	0.0060 (15)	0.0004 (16)

### Geometric parameters (Å, °)

**Table:**

N1—C12	1.305 (3)	C7—C8	1.417 (4)
N1—C13	1.396 (3)	C8—C9	1.371 (4)
N2—C1	1.303 (3)	C8—H8	0.9300
N2—C12	1.402 (3)	C9—C10	1.393 (4)
O1—C3	1.342 (3)	C9—H9	0.9300
O1—H1	0.8200	C10—C11	1.360 (4)
S1—C14	1.734 (3)	C10—H10	0.9300
S1—C12	1.749 (3)	C11—H11	0.9300
C1—C2	1.421 (4)	C13—C14	1.399 (4)
C1—H1A	0.9300	C13—C18	1.400 (4)
C2—C3	1.410 (4)	C14—C15	1.399 (4)
C2—C7	1.446 (3)	C15—C16	1.380 (4)
C3—C4	1.409 (4)	C15—H15	0.9300
C4—C5	1.348 (4)	C16—C17	1.386 (4)
C4—H4	0.9300	C16—H16	0.9300
C5—C6	1.424 (4)	C17—C18	1.368 (4)
C5—H5	0.9300	C17—H17	0.9300
C6—C11	1.407 (4)	C18—H18	0.9300
C6—C7	1.417 (4)		
			
C12—N1—C13	110.0 (2)	C8—C9—H9	119.4
C1—N2—C12	118.0 (2)	C10—C9—H9	119.4
C3—O1—H1	109.5	C11—C10—C9	119.2 (3)
C14—S1—C12	88.99 (14)	C11—C10—H10	120.4
N2—C1—C2	123.5 (3)	C9—C10—H10	120.4
N2—C1—H1A	118.3	C10—C11—C6	121.2 (3)
C2—C1—H1A	118.3	C10—C11—H11	119.4
C3—C2—C1	119.8 (3)	C6—C11—H11	119.4
C3—C2—C7	118.6 (2)	N1—C12—N2	126.0 (3)
C1—C2—C7	121.5 (2)	N1—C12—S1	116.2 (2)
O1—C3—C2	122.2 (3)	N2—C12—S1	117.9 (2)
O1—C3—C4	117.1 (3)	N1—C13—C14	115.3 (3)
C2—C3—C4	120.8 (3)	N1—C13—C18	124.4 (3)
C5—C4—C3	120.1 (3)	C14—C13—C18	120.3 (3)
C5—C4—H4	119.9	C15—C14—C13	120.5 (3)
C3—C4—H4	119.9	C15—C14—S1	130.0 (2)
C4—C5—C6	122.5 (3)	C13—C14—S1	109.5 (2)
C4—C5—H5	118.7	C16—C15—C14	118.0 (3)
C6—C5—H5	118.7	C16—C15—H15	121.0
C11—C6—C7	120.3 (3)	C14—C15—H15	121.0
C11—C6—C5	121.3 (3)	C15—C16—C17	121.3 (3)
C7—C6—C5	118.4 (3)	C15—C16—H16	119.3
C8—C7—C6	116.9 (2)	C17—C16—H16	119.3
C8—C7—C2	123.6 (2)	C18—C17—C16	121.4 (3)
C6—C7—C2	119.5 (2)	C18—C17—H17	119.3
C9—C8—C7	121.1 (3)	C16—C17—H17	119.3
C9—C8—H8	119.4	C17—C18—C13	118.5 (3)
C7—C8—H8	119.4	C17—C18—H18	120.8
C8—C9—C10	121.3 (3)	C13—C18—H18	120.8
			
C12—N2—C1—C2	178.8 (2)	C9—C10—C11—C6	−0.5 (4)
N2—C1—C2—C3	−1.2 (4)	C7—C6—C11—C10	0.9 (4)
N2—C1—C2—C7	179.9 (2)	C5—C6—C11—C10	−179.1 (3)
C1—C2—C3—O1	0.1 (4)	C13—N1—C12—N2	−179.4 (2)
C7—C2—C3—O1	179.1 (2)	C13—N1—C12—S1	0.8 (3)
C1—C2—C3—C4	179.8 (2)	C1—N2—C12—N1	8.5 (4)
C7—C2—C3—C4	−1.3 (4)	C1—N2—C12—S1	−171.67 (19)
O1—C3—C4—C5	179.8 (2)	C14—S1—C12—N1	−0.1 (2)
C2—C3—C4—C5	0.1 (4)	C14—S1—C12—N2	−180.0 (2)
C3—C4—C5—C6	1.0 (4)	C12—N1—C13—C14	−1.3 (3)
C4—C5—C6—C11	179.3 (3)	C12—N1—C13—C18	179.0 (3)
C4—C5—C6—C7	−0.8 (4)	N1—C13—C14—C15	179.9 (2)
C11—C6—C7—C8	−0.7 (3)	C18—C13—C14—C15	−0.4 (4)
C5—C6—C7—C8	179.3 (2)	N1—C13—C14—S1	1.2 (3)
C11—C6—C7—C2	179.5 (2)	C18—C13—C14—S1	−179.1 (2)
C5—C6—C7—C2	−0.5 (3)	C12—S1—C14—C15	−179.1 (3)
C3—C2—C7—C8	−178.3 (2)	C12—S1—C14—C13	−0.56 (19)
C1—C2—C7—C8	0.7 (4)	C13—C14—C15—C16	0.5 (4)
C3—C2—C7—C6	1.4 (3)	S1—C14—C15—C16	178.9 (2)
C1—C2—C7—C6	−179.6 (2)	C14—C15—C16—C17	−0.3 (4)
C6—C7—C8—C9	0.1 (4)	C15—C16—C17—C18	0.1 (4)
C2—C7—C8—C9	179.9 (2)	C16—C17—C18—C13	0.0 (4)
C7—C8—C9—C10	0.3 (4)	N1—C13—C18—C17	179.9 (2)
C8—C9—C10—C11	−0.1 (4)	C14—C13—C18—C17	0.2 (4)

### Hydrogen-bond geometry (Å, °)

**Table:**

*D*—H···*A*	*D*—H	H···*A*	*D*···*A*	*D*—H···*A*
C16—H16···N1^i^	0.93	2.63	3.389 (4)	140.
O1—H1···N2	0.82	1.85	2.576 (2)	148.

Symmetry codes: (i) *x*+1/2, −*y*+1/2, *z*+1/2.
